# Role of Extracellular Vesicles in the Pathogenesis of Brain Metastasis

**DOI:** 10.1002/jex2.70051

**Published:** 2025-05-06

**Authors:** Muhammad Izhar, Maciej S. Lesniak

**Affiliations:** ^1^ Department of Neurosurgery Massachusetts General Hospital, Harvard Medical School Boston Massachusetts USA; ^2^ Department of Neurosurgery Stanford University School of Medicine Stanford California USA; ^3^ Department of Neurological Surgery Lou and Jean Malnati Brain Tumor Institute, Robert H. Lurie Comprehensive Cancer Center, Feinberg School of Medicine, Northwestern University Chicago Illinois USA

**Keywords:** blood–brain barrier, brain metastasis, EVs, exosomes, extracellular vesicles

## Abstract

Extracellular vesicles (EVs) are small particles released by various cells, including cancer cells. They play a significant role in the development of different cancers, including brain metastasis. These EVs transport biomolecular materials such as RNA, DNA, and proteins from tumour cells to other cells, facilitating the spread of primary tumours to the brain tissue. EVs interact with the endothelial cells of the blood–brain barrier (BBB), compromising its integrity and allowing metastatic cells to pass through easily. Additionally, EVs interact with various cells in the brain's microenvironment, creating a conducive environment for incoming metastatic cells. They also influence the immune system within this premetastatic environment, promoting the growth of metastatic cells. This review paper focuses on the research regarding the role of EVs in the development of brain metastasis, including their impact on disrupting the BBB, preparing the premetastatic environment, and modulating the immune system. Furthermore, the paper discusses the potential of EVs as diagnostic and prognostic biomarkers for brain metastasis.

**Abbreviations**
ACE: angiotensin‐I‐converting enzyme
AGO2: Argonaute 2
CCL1: C‐C motif chemokine ligand 1
CCL2: cytokine chemokine (C‐C motif) ligand 2
CXCL: C‐X‐C motif chemokine ligand 1
EPB41L5: erythrocyte membrane protein band 4.1 like 5
GLUT: glucose transporter
GPC1: Glypican 1
Iba1: ionized calcium‐binding adaptor molecule 1
MET: mesenchymal epithelial transmission
NF‐κB: nuclear factor‐kappa B
NOTCH: NOTCH receptor/no full form
NUMB: NUMB endocytic adaptor protein/no full form
PCSK9: proprotein convertase subtilisin/kexin type 9
PD‐L1: programmed cell death ligand 1
PDPK1:  3‐phosphoinositide‐dependent protein kinase‐1
PKM: pyruvate kinase
PTGS2: prostaglandin‐endoperoxide synthase 2
STAT: signal transducer and activator of transcription
TGFBI: transforming growth factor, beta‐induced
Tnf: tumour necrosis factor

## 1 Introduction

**FIGURE 1:**
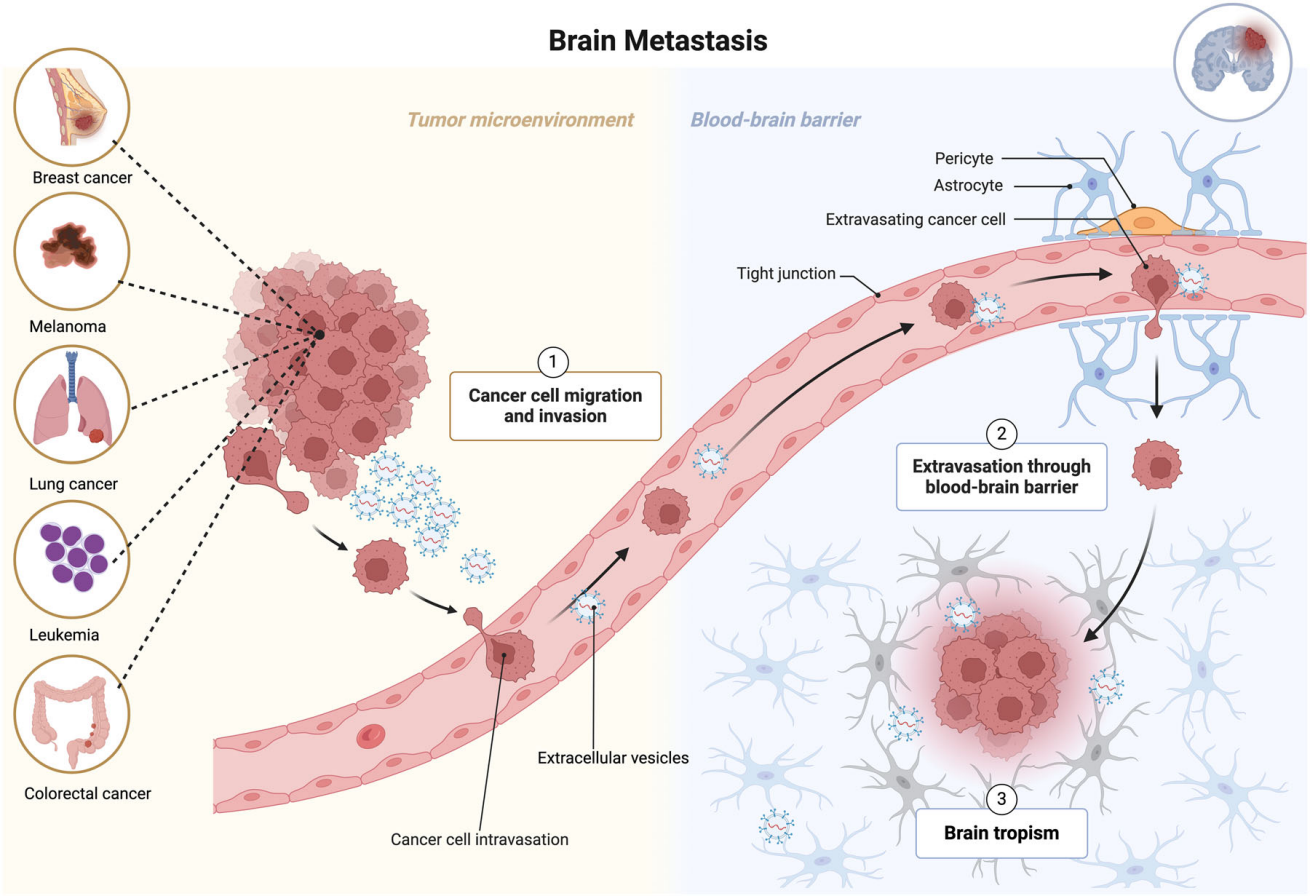
Mechanism of brain metastasis. The metastatic cells migrate from the primary sites most commonly breast, skin and lung, and invade the blood vessels entering the bloodstream. These brain tropic metastatic cells flow to the brain microvessels, extravasate through disrupted BBB and enter the premetastatic niche of the brain.

Brain metastatic cancer, often referred to as brain metastases (BM), occurs when cancer cells spread from their primary site (lung, breast or skin) to the brain (Fares et al. 2020). It is the most common malignancy of the central nervous system (CNS). An estimated 20% of patients with cancer will develop BM (Nayak et al. 2012; Barnholtz‐Sloan et al. 2004). Lung cancer accounts for 20%–56% of these cases, followed by breast cancer (BC) at 5%–20% and melanoma at 7%–16% (Nayak et al. 2012; Barnholtz‐Sloan et al. 2004; Sperduto et al. 2010). Lung cancer has the highest rate of spreading to the brain across all patient genders, making it the predominant cause of BM in men. For women, however, BC is the most frequent source of BM (Achrol et al. 2019). Despite advances in treatment that include a variety of approaches, those with CNS metastases generally face grim prognoses, with fewer than 10% surviving beyond 2 years post‐diagnosis, alongside significantly diminished quality of life (Hall et al. 2000). A crucial phase in the progression of BM involves the migration of cancer cells across the blood–brain barrier (BBB) (Arshad et al. 2011; Bos et al. 2010), composed of endothelial cells and other supporting cells (Winkler et al. 2011; Ballabh et al. 2004) (Figure 1). The BBB acts as a selective barrier, impeding the uncontrolled movement of substances. A distinctive characteristic of BM is the compromise of the BBB's integrity (Lee et al. 2003). Cancer cells manage to identify and bind to elements of the vascular wall, initiating a series of events that include breaking through the BBB, invading the brain, and initiating tumour growth in this new location (Nicolson 1988; Orr et al. 2000) (Figure 1).

Extracellular vesicles (EVs), including exosomes, play a significant role in various cancer‐related processes (Chang et al. 2021) by facilitating intercellular communication via transporting proteins, mRNA and microRNA (miRNA) between cells. Moreover, EVs can help in the diagnosis and prognosis of brain cancer. While the involvement of EVs in promoting metastasis is evident, their specific functions in the context of BM remain less understood. In this review, we will discuss the role of EVs in BM.

## 2 Blood–Brain Barrier

The BBB is made up of brain microvascular endothelial cells (BMECs), pericytes, astrocytes, endothelial basement membranes, and neighbouring neurons (Di Lorenzo and Ahluwalia 2017) (Figure 1). The passage of substances through the BBB is a tightly controlled process. Endothelial cells forming the BBB are joined by tight junctions that limit passive diffusion (Berghoff and Preusser 2018; Izhar et al. 2024).

Pericytes also contribute to the stability of the BBB by regulating the expression of tight and adherent junctions, limiting transcytosis, producing components of the extracellular matrix (ECM), and initiating new blood vessel formation. Furthermore, the basal membrane, which comprises endothelial cells and pericytes, adds a layer of protection (Obermeier et al. 2013). Astrocytic foot processes closely interact with the basal membrane and link the BBB to the neuronal brain parenchyma, thereby aiding in regulating diffusion restrictions across the BBB (Gee and Keller 2005). The paracellular route across tight junctions plays a vital role in facilitating the passive diffusion of water, gases, and lipid‐soluble molecules (Berghoff and Preusser 2018). Physiologically, molecules exceeding 400 Da cannot cross the BBB through passive diffusion, as their size is too large (Fares et al. 2020). The process of transmigration is also influenced by the charge and chemical properties of the molecules (hydrophilic/lipophilic) (Fares et al. 2020). Solutes and nutrients can traverse the BBB transcellular through passive diffusion or active transport (Berghoff and Preusser 2018). Lipid‐soluble molecules can pass through passively, while glucose and amino acids may require carrier‐mediated transporters and metabolic energy to cross the BBB (Berghoff and Preusser 2018). The process of forming BM is hindered by the rate‐limiting step of crossing the BBB. Two potential pathways for this transit have been identified: the paracellular pathway, which occurs between endothelial cells, and the transcellular pathway, which involves movement through the endothelial cells. Both in vitro and in vivo studies have shown that cellular diapedesis can happen by forming a gap in the paracellular route or by creating a transcellular pore (Fares et al. 2020; Arvanitis et al. 2020).

## 3 Extracellular Vesicles

EVs are recognized for their distinctive composition of lipids, proteins, and nucleic acids, which mirrors that of their cell and tissue of origin (Yates et al. 2022). They are classified based on their biogenesis. The smallest EVs, traditionally called exosomes (30–150 nm), are generated through the endolysosomal pathway, formed by the invagination of endosomes, and stored in multivesicular bodies before being released via exocytosis (Yates et al. 2022). Microvesicles (MVs), called microparticles or ectosomes, are larger (100–1000 nm) and bud directly from the plasma membrane. Apoptotic bodies, the largest EVs (1000–5000 nm), are released following programmed cell death (Yates et al. 2022).

EVs play versatile and critical roles in various physiological processes, spanning reproductive biology (Yates et al. 2022; Park et al. 2011; Ronquist 2012; Rooney et al. 1993; Palmerini et al. 2003), CNS function (Yates et al. 2022; Paolicelli and Ferretti 2017; Mayo, and Bearden, 2015), musculoskeletal health (Yates et al. 2022; Choi et al. 2016; Forterre et al. 2014; Le Bihan et al. 2012; Coenen‐Stass et al. 2016; Guescini et al. 2015; Deng et al. 2015; Huynh et al. 2016), and immune (Hong 2018, Zhou et al. 2020; Timár et al. 2013; Lim et al. 2015; Nolan et al. 2008; Nolte‐‘t Hoen et al. 2009; Théry et al. 2002), cardiovascular (Good et al. 2020; Pironti et al. 2015), and renal systems (Street et al. 2011; Hiemstra et al. 2014; Jella et al. 2016). Besides cell‐to‐cell communications, they can carry nutrients (Hendricks et al. 2021), and cellular garbage (Johnstone 1992), and initiate mineral formation (Shapiro et al. 2015). Various cell types produce them, such as erythrocytes, platelets, lymphocytes, dendritic cells (DCs), adipocytes, fibroblasts, brain cells, stem cells, and cancer cells (Blott and Griffiths 2002). Bodily fluids like blood, plasma, urine, cerebrospinal fluid (CSF), milk, amniotic fluid, malignant ascites, saliva, and synovial fluid contain these substances. EVs reflect the unique characteristics of the cells from which they are derived (Yates et al. 2022). These vesicles encase various biomolecules specific to the parent cells' physiological state (Hallal et al. 2022; Yáñez‐Mó et al. 2015). These biomolecules include a variety of proteins such as transcription factors, surface receptors, heat‐shock proteins, lipids, and various nucleic acids, like DNA, mRNA, miRNA, and other noncoding RNAs (Hallal et al. 2022; Yáñez‐Mó et al. 2015). These biomolecules provide a wealth of information about the parent cells, making EVs a promising tool for diagnostic and therapeutic applications (Tkach and Théry 2016; Thakur et al. 2022; Aqil et al. 2017; Dai et al. 2020). These EVs have been known to play a role in cancer progression, immune suppression in the tumour microenvironment (TME), angiogenesis, and metastasis (Ghaemmaghami et al. 2020; Hoshino et al. 2015; Costa‐Silva et al. 2015).

## 4 Preparation of the Pre‐Metastatic Niche for the Incoming Metastatic Cells

David Lyden first introduced the concept of the pre‐metastatic niche (PMN) (Kaplan et al. 2005). This concept involves the creation of a specialized microenvironment in a distant organ that initially lacks cancer cells. This environment is shaped by the primary tumour, which emits various tumour factors and EVs, which travel to secondary organs, altering the ECM, modifying vascular permeability (Peinado et al. 2017; Rodrigues et al. 2019; Wu et al. 2021; Gener Lahav et al. 2019), and changing the immune response by merging with local cells and transferring their load of proteins, diverse metabolites, or genetic materials (Peinado et al. 2017; Rodrigues et al. 2019; Wu et al. 2021; Gener Lahav et al. 2019). These PMNs create the ideal conditions for the incoming metastatic cells to survive and proliferate (Peinado et al. 2017; Valadi et al. 2007; Shibue and Weinberg 2011; Sleeman 2012).

**FIGURE 2:**
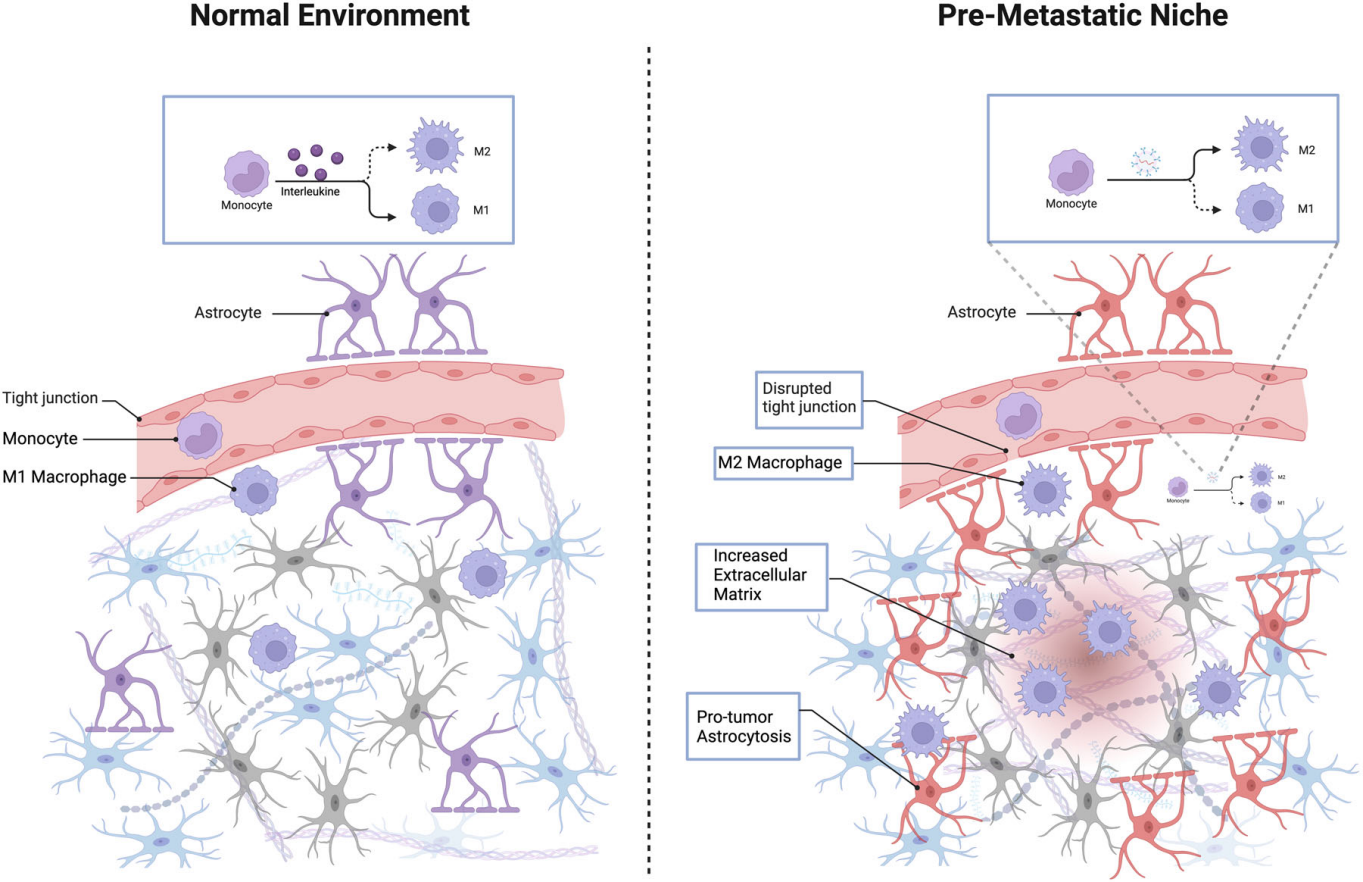
Comparison of normal brain microenvironment and pre‐metastatic niche. EVs released from primary tumours induce M2 polarization of monocytes (or macrophages) in the pre‐metastatic niche of the brain, promoting metastatic growth and reducing anti‐tumour immunity. Moreover, the EVs also modulate the extracellular matrix (ECM), decrease glucose uptake and induce pro‐metastatic astrocytosis, preparing the brain's pre‐metastatic niche for metastatic cells.

Astrocytes, the supportive cells in the brain, emit EVs that reduce the expression of the phosphatase and tensin homologue (PTEN) gene, a crucial tumour suppressor, particularly in BM (Zhang et al. 2015). Zhang et al. (Zhang et al. 2015) demonstrated that in‐vivo BC cell lines (MDA‐MB‐231, HCC1954, BT474, and MDA‐MB‐435) initially possessing normal PTEN levels exhibit a loss of PTEN expression upon infiltration into the brain of Swiss nude mice. Interestingly, PTEN levels remain unchanged following tumour dissemination to other organs (Zhang et al. 2015). Furthermore, brain metastatic cells experiencing decreased PTEN expression regain normal PTEN levels upon exiting the brain microenvironment (Zhang et al. 2015). This phenomenon was attributed to the release of small EVs by astrocytic cells of PMN in the brain, which carry PTEN‐targeting miR‐19a molecules transferred to BC brain metastatic cells, resulting in PTEN loss (Zhang et al. 2015). In‐vitro, when the metastatic cells were co‐cultured with primary glia (>90 % astrocytes), there was a significant decrease in PTEN mRNA and protein (Zhang et al. 2015). Additionally, the loss of PTEN in brain metastatic tumour cells prompted an increase in the secretion of the CCL2 chemokine and the recruitment of Iba1‐expressing myeloid cells, as shown in (Figure 2), thereby intensifying the brain metastatic process through enhanced tumour cell proliferation and reduced apoptosis (Zhang et al. 2015).

The EVs from BC cells re‐programme the glucose consumption in niche tissue and promote metastasis (Fong et al. 2015). Brain astrocytes take up the miR‐122‐containing EVs released by incoming metastatic cells, reducing the level of PKM and GLUT in astrocytes (Fong et al. 2015). This decrease in glucose uptake by astrocytes preserves glucose for the incoming metastatic cells (Fong et al. 2015). In vitro, brain astrocytes co‐cultured with high‐miR‐122 EVs from the MDA‐MB‐231 BC cell line had significantly increased miR‐122 in the astrocytes, resulting in significantly decreased expression of PKM2 and GLUT1 (Fong et al. 2015). In vivo, MDA‐MB‐231‐HM cells were injected into mice intra‐cardially following pre‐conditioning with injections of high miR‐122‐EVs obtained from xenograft tumours of MDA‐MB‐231‐HM. The treatment group exhibited significant metastatic colonization to the lungs and brain compared to the control group (Fong et al. 2015). Moreover, to study the effect of inhibition of miR‐122, the mice with established MDA‐MB‐231‐HM xenograft tumours were treated with anti‐miR‐122 and found to have a lower incidence of metastasis to the brain and lungs compared to control groups (Fong et al. 2015).

The EVs from derived BMECs in PMN improve the survival of the recipient small cell lung cancer cells (SCLC) in the brain and their resistance to apoptosis (Xu et al. 2019). An in vitro study showed that EVs from human BMEC upregulated the expression of S100A16 in SCLC cells (NCL‐H209 and NCL‐H446 cell lines) (Xu et al. 2019). The S100A16 translocates from the cytoplasm to the nucleus of SCLC cells, triggering the expression of prohibitin (PHB)‐1 (Xu et al. 2019). PHB‐1, a protein in the mitochondrial inner membrane, is vital in forming mitochondrial permeability transition pores (mPTPs), depolarizing the mitochondrial membrane, and generating reactive oxygen species (Artal‐Sanz and Tavernarakis 2009). PHB complex in the inner mitochondrial membrane stabilizes the mitochondrial membrane potential and morphology, which are essential for mitochondrial function and cell survival (Peng et al. 2015). The primary way PHB‐1 provides protection is by inhibiting the intrinsic apoptotic pathway (Li et al. 2015). This is achieved by preserving mitochondrial membrane potential, preventing the release of cytochrome c and inhibiting the activation of caspase‐3 (Chowdhury et al. 2013). Moreover, PHB‐1 contributes to this protective effect by increasing the transcription and translation of anti‐apoptotic genes such as Bcl2 and Bclxl (Chowdhury et al. 2013; Chowdhury et al. 2011). The protective effects of BMEC EVs were reversed by the S100A16 knockdown in SCLC cells, delivery of inhibitory S100A16 and PHB1 siRNAs to SCLC cells and the use of exosome release and biogenic blockers GW4869 (Xu et al. 2019).

BC‐releasing EVs remodel the brain microenvironment to foster its metastasis (Rodrigues et al. 2019). Rodrigues et al. (2019) reported that cell migration–inducing and hyaluronan‐binding protein (CEMIP)–containing EVs from brain metastatic BC cells (231 BrT1) alter the brain's PMN to promote brain metastasis. In ex vivo, an organotypic brain slice culture system was employed for the study. Brain slices were pre‐treated with 5 µg of EVs derived from brain‐tropic 231‐BR (231 BrT1), lung‐tropic 4175 (231 LuT1), bone‐tropic 1833 (231 BoT1) or parental MDA‐MB‐231 (231 Parental) human BC metastatic cells for 2 consecutive days. Subsequently, fluorescently labelled 231 BrT1 cancer cells were added, and tumour cell colonization was assessed 3 days later. Pre‐treatment with 231 BrT1‐derived EVs resulted in a four‐fold increase in colonizing 231 BrT1 cell numbers compared to phosphate‐buffered saline (PBS) and at least a two‐fold increase compared to pre‐treatment with 231 parental, lung‐metastatic or bone‐metastatic EVs. In contrast, pre‐treatment with non‐brain‐tropic EVs did not significantly enhance cancer cell growth compared to PBS (Rodrigues et al. 2019). Furthermore, 3 days after tumour cell addition, invading 231 BrT1 cells were quantified in transversal sections of brain slices pre‐treated with either 231 BrT1‐derived EVs or 231 parental‐derived EVs. The results showed that pre‐treatment with 231 BrT1 EVs increased 231 BrT1 cell invasiveness three‐fold compared to pre‐treatment with 231 parental‐derived EVs or PBS (Rodrigues et al. 2019). In vivo, mice were administered 10 µg of 231 BrT1 CEMIP wild‐type (WT) or CEMIP knockout‐derived EVs every other day for 3 weeks before the intra‐cardiac injection of 231 BrT1 GFP‐luciferase+ cells. Pre‐treatment with 231 BrT1 CEMIP WT‐derived EVs significantly enhanced brain metastasis compared to CEMIP knockout‐derived EV pre‐treatments at Weeks 1 and 2 post‐injection. Brain lesion quantification revealed a higher number of metastatic foci and increased metastatic burden in mice pre‐treated with CEMIP WT EVs compared to PBS or CEMIP knockout EVs pre‐treated groups (Rodrigues et al. 2019). Depleting CEMIP in tumour cells hindered brain metastasis by disrupting the invasion process and the tumour cells' interaction with brain blood vessels (Rodrigues et al. 2019). These effects were reversed when the brain microenvironment was pre‐conditioned with CEMIP‐positive EVs (Rodrigues et al. 2019). Additionally, when brain endothelial and microglial cells were treated with CEMIP‐positive EVs, endothelial cell branching and inflammation in the perivascular niche were induced (Rodrigues et al. 2019). Inflammation was associated with an increase in the expression of pro‐inflammatory cytokines encoded by Ptgs2, Tnf and Ccl/Cxcl genes (Rodrigues et al. 2019), which are known to facilitate brain vascular re‐modelling and metastasis (Doron et al. 2019).

Hoshino et al. (Hoshino et al. 2015) demonstrated that the EVs released from one organotropic metastatic cell are several‐fold localized in that specific organ compared to those released from another type of organotropic metastatic cells. He collected EVs from different sublines of the metastatic BC cell line (MDA‐MB‐231), that is, 4175‐LuT, 1833‐BoT and 831‐BrT, which colonize lung, bone and brain, respectively. He then injected these EVs into C57BL/6 female nude mice and found that the EVs were localized severalfold in their trophic organs. He observed that the EVs from 831‐BrT sublines were preferentially localized to the brain with a more than four‐fold increase compared to 1833‐BoT and 4175‐LuT EVs. Furthermore, he found that different integrins on EVs from different sublines were abundant (Hoshino et al. 2015). Integrin ITG β3 was abundant in the EVs released from brain‐tropic cells (Hoshino et al. 2015). Moreover, he demonstrated that organotropic EVs increase vascular permeability and modulate metastatic organotropism. Integrin knockdown in organotropic EVs was shown to reduce organotropic metastasis. So, integrin on the surface of EVs from BC could be potentially used to predict organ‐specific metastasis (Hoshino et al. 2015).

EVs can alter astrocyte behaviour, creating a microenvironment conducive to metastatic cell proliferation (Rojiani et al. 2015; Wu et al. 2015; Mendes et al. 2005; Mendes et al. 2007). It is well recognized that matrix metalloproteinases (MMPs) and their natural inhibitors, tissue inhibitors of MMPs (TIMPs), play significant roles in tumour progression and metastasis (Roy et al. 2020; Fang et al. 2000; Overall and Kleifeld 2006; Harper and Moses 2006). By influencing the ECM, these enzymes and inhibitors initiate various signalling pathways that foster a tumour‐promoting microenvironment (Peinado et al. 2017; Liu and Cao 2016) (Figure 2). Numerous studies have highlighted the critical role of MMPs and TIMPs in establishing a conducive PMN for the growth of incoming cancer cells in the brain (Rojiani et al. 2015; Wu et al. 2015; Mendes et al. 2005; Mendes et al. 2007). Morad et al. (2020) demonstrated that the EVs from BC brain metastatic cells (MDA‐MB‐231 cell line) interact with human brain astrocytes, decreasing the expression level of TIMP‐2 in vitro and in vivo, making the supportive PMN. He found that the miR‐301a‐3P in the EVs downregulates TIMP‐2 in the astrocytes of PMN.

Rigg et al. (2023) reported that the EVs released from human melanoma brain metastasis (MBM) cell lines (H1, H2) create a brain metastatic niche, enhancing the initiation and growth of MBM. He found that these EVs carry miR‐146a‐5p to normal human astrocytes (NHA), resulting in their activation (Figure 2) and cytokines release. The miR‐146a‐5p increases the expression levels of NOTCH and downstream proteins in NHA through the downregulation of NUMB, releasing cytokine IL‐6, IL‐8, MCP‐1 (CCL2) and CXCL1 (Rigg et al. 2023). The cytokines contribute to MBM growth (Rigg et al. 2023; Zhang et al. 2018; Wasilewski et al. 2017; Fares et al. 2021). For the first time, Rigg et al. (2023) reported the role of MBM‐secreted EVs in the activation of astrocytes. The knockdown of miR‐146a‐5p in the MDA‐MB‐231 cell line showed decreased tumour burden and increased mice survival (Rigg et al. 2023).

The EVs can transform non‐metastatic cancer cells into metastatic cells (Camacho et al. 2013). Camacho et al. (2013) demonstrated that EVs originating from BC brain metastatic MDA‐MB‐231 cells carry metastasis‐associated proteins and miRNAs to non‐metastatic cells of BC, enhancing their adhesive and invasive properties.

The EVs derived from BC brain metastatic cells may activate astrocytes in PMN via transferring miR‐1290 and mir‐1246 to them (Sirkisoon et al. 2022) (Figure 2). In vitro, the miR‐1290 and mir‐1246 in MDA‐MB‐231 cells derived EVs targeted Forkhead Box A2 (FOXA2) in  E6/E7/hTERT immortalized human astrocytes and decreased their expression level (Sirkisoon et al. 2022). The FOXA2 is a direct transcriptional repressor of ciliary neurotrophic factor (CNTF) (Sirkisoon et al. 2022). Therefore,  the reduced FOXA2 led to increased CNTF expression and secretion, which resulted in astrocyte activation and BC aggressiveness (Sirkisoon et al. 2022). In vivo,  a brain metastatic human BC cell line derived from HER2‐enriched SKBR3 cells (SKBRM cells) and miR‐1290 overexpressing astrocytes were stereotactically implanted into the right frontal lobe of athymic female mice brain and was demonstrated that the astrocytes overexpressing miR‐1290 promoted the intra‐cranial colonization and growth of SKBRM cells (Sirkisoon et al. 2022).

## 5 Extracellular Vesicles From Primary Tumour Disrupt the Integrity of the Blood–Brain Barrier

**FIGURE 3:**
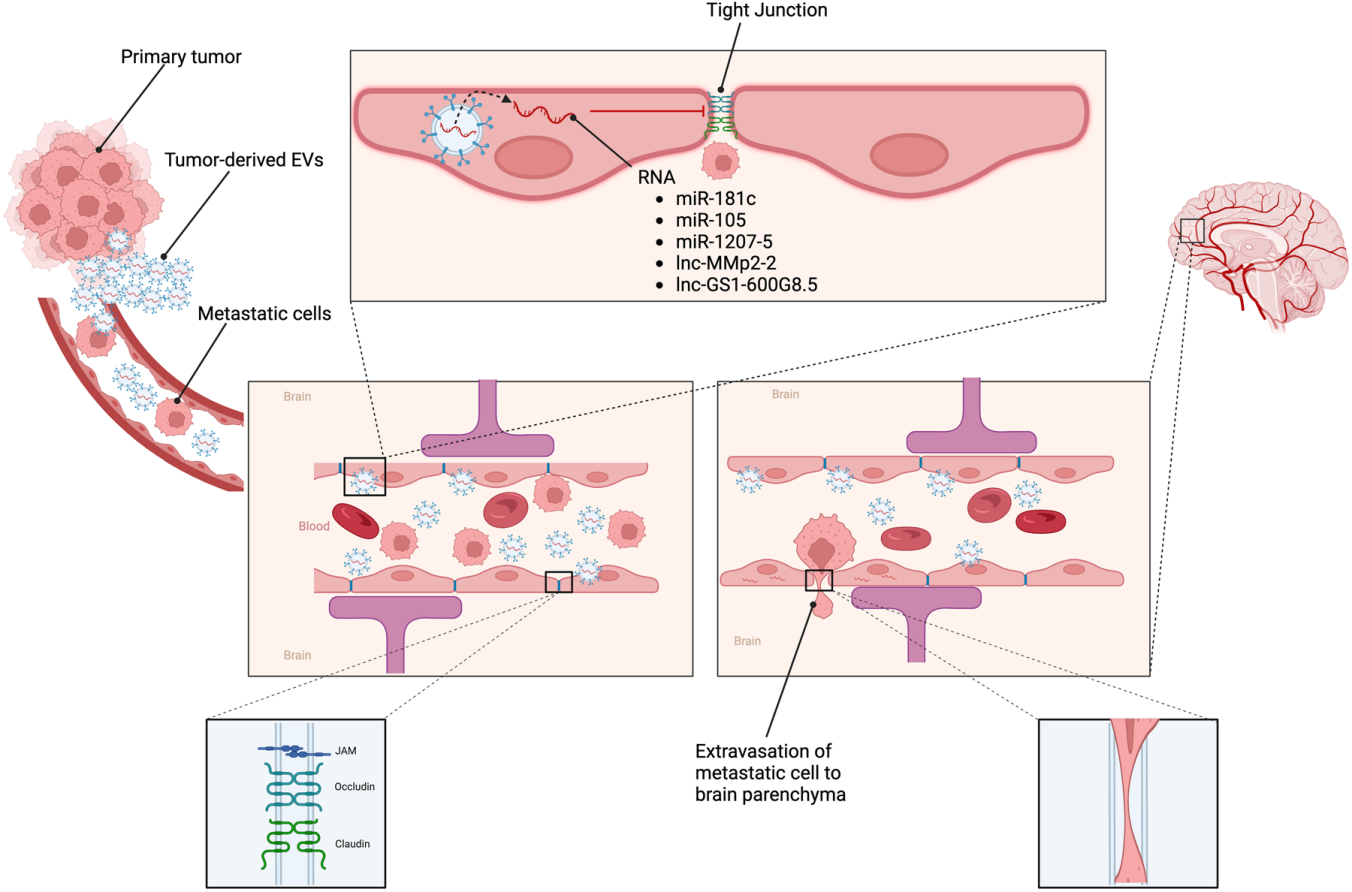
Extracellular vesicles induce disruption of tight junctions of BBB. The extracellular vesicles (EVs) are taken up by endothelial cells of the blood–brain barrier (BBB), which release miRNA and/or long noncoding RNA. The miRNAs or lnc‐RNAs then downregulate or inhibit the mRNA that translates into the protein of tight junctions, thereby disrupting the BBB and allowing metastatic cells to enter the brain parenchyma.

The CNS does not have a lymphatic system, so brain metastatic cells reach the brain parenchyma through blood, breaching the BBB (Figure 3) (Wilhelm et al. 2013). The critical step for metastatic cells to cross the BBB is adhesion to the endothelium (Achrol et al. 2019), influenced by the EVs released from metastatic cells (Fazakas et al. 2021). Fazakas et al. (2021) employed atomic force microscopy to analyse the de‐adhesion strength between breast adenocarcinoma cells and the brain's endothelial cells. He found that human microvascular cerebral endothelial cells (hCMEC/D3) pre‐treated with EVs derived from BC MDA‐MB‐231 cells exhibited a reduced deadhesion strength compared to untreated hCMEC/D3 cells.

The human BBB is essential to the brain's protection system (Fares et al. 2020). Tight junctions play a crucial role in maintaining the BBB's low permeability (Berghoff and Preusser 2018). These tight junctions consist of specific proteins found in endothelial cells, including Claudin‐5, Occludin and ZO‐1 (Haseloff et al. 2015; Liebner et al. 2000). On the other hand, N‐cadherin is a glycoprotein that facilitates robust cell–cell adhesion through five extracellular cadherin repeats (Haseloff et al. 2015; Liebner et al. 2000; Salomon et al. 1992). It is mainly found on the apical and basal membranes (Haseloff et al. 2015). Both tight junction proteins and N‐cadherin contribute to cell polarity by closely interacting with the actin cytoskeletal network (Haseloff et al. 2015; Liebner et al. 2000). Dysregulation of these critical molecules is linked to the disruption of the BBB in many human diseases (Zhao et al. 2015).

Tominaga et al. (2015) found that the EVs released from BC brain metastatic cells dysregulated these tight junction proteins and N‐Cadherin, disrupting BBB (Figure 3). The miRNA‐181c in these EVs causes the downregulation of the 3‐phosphoinositide‐dependent protein kinase‐1 (PDPK1) in endothelial cells (Tominaga et al. 2015). PDPK1 localizes the tight junction–related protein, N‐cadherin and actin (Tominaga et al. 2015). These proteins were found at the cellular membrane in control and PDPK1 siRNA–treated endothelial cells, while their localization was dysregulated in PDPK1 siRNA–treated endothelial cells (Tominaga et al. 2015). PDPK1 has been reported to be an upstream protein of the cofilin phosphorylation (Lyle and Griendling 2006; Higuchi et al. 2008). Cofilin, a family of actin‐binding proteins, disassembles actin filaments and is activated through dephosphorylation (Tominaga et al. 2015). In an in vitro study, the brain endothelial cells (MBT24‐H) cultured with MDA‐MB‐231‐luc‐D3H2LN BC cells–derived EVs were shown to have reduced cofilin phosphorylation (Tominaga et al. 2015). In vivo, the systemic administration of BC brain metastatic D3H2LN cells derived EVs to mice promoted the metastasis of MDA‐MB‐231‐luc‐D3H2LN cell lines preferentially to the brain via disruption of BBB (Tominaga et al. 2015).

The miR‐105 is enriched in EVs released from MDA‐MB‐231 metastatic BC cells compared to EVs released from MCF‐10A non‐cancerous mammary epithelial line (Zhou et al. 2014). In vitro EVs from MDA‐MB‐231 carry miR‐105 to primary human microvascular endothelial cells (HMVECs). The miR‐105 targets the binding sites in the 3′UTR of human Zonula Occludens‐1 (ZO‐1) in HMVECs, reducing the expression of ZO‐1, a significant component of the tight junction, at mRNA and protein levels (Zhou et al. 2014). This reduced level of ZO‐1 results in increased vascular permeability, promoting BC metastasis (Zhou et al. 2014). An in vivo study in a mouse model also showed that miR‐105 increased vascular permeability and enhanced metastasis, and its inhibition suppressed metastasis and restored vascular integrity (Zhou et al. 2014).

Wu et al. (2021) reported that EVs derived from TGF‐β1‐mediated non‐small cell lung cancer (NSCLC) A549 cell line disrupt the tight junctions and the integrity of BBB in in vitro and in vivo studies, promoting brain metastasis (Figure 3). These EVs contain lnc‐MMP2‐2, downregulating the tight junction proteins when incorporated into non‐foetal‐derived human brain microvascular endothelial cells (HBMECs). The lnc‐MMp2‐2 increases the permeability of BBB by competing with the miR‐1207‐5, which modulates the EPB41L5 derepression. In vitro, the knockdown and overexpression of EPB41L5 in HBMECs confirmed that EPB41L5 significantly promoted endothelial‐to‐mesenchymal transition, disrupted tight junctions and induced HBMECs’ monolayer permeability (Wu et al. 2021). To further explore the impact of lnc‐MMP2‐2 on BM in NSCLC, an in vivo mouse model of BM was established (Wu et al. 2021). The model was created by injecting A549 cells stably transfected with either a control or shlnc‐MMP2‐2 lentivirus, then treated with or without TGF‐β1 (Wu et al. 2021). The results showed that the knockdown of lnc‐MMP2‐2 significantly decreased the incidence of BM, indicating the potential of lnc‐MMP2‐2 as a therapeutic target for NSCLC BM (Wu et al. 2021).

BMECs take up EVs from highly brain metastatic cells of BC (MDABR3 cells), decreasing transepithelial/transendothelial electrical resistance (TEER) and increasing BBB permeability (Figure 3) (Lu et al. 2020). These EVs, originating from highly metastatic MDABR3 cells, enhanced the invasion of BC cells in the BBB model (Lu et al. 2020). The long non‐coding RNA GS1‐600G8.5 was found at higher levels in the  MDABR3 cells and their EVs than those from less metastatic samples (MDA231 cells) (Lu et al. 2020). In vitro, silencing GS1‐600G8.5 markedly reduced the BBB‐disruptive effects of the EVs (Lu et al. 2020). EVs from MDA231 cells, deficient in GS1‐600G8.5, did not facilitate the infiltration of MDA231 cancer cells through the BBB but the EVs from highly brain metastatic MDABR3 cells, rich in GS1‐600G8.5, facilitated the infiltration of MDA231 cancer cells through the BBB (Lu et al. 2020). Additionally, in vitro,  brain capillary epithelial cells (BMECs) exposed to GS1‐600G8.5‐depleted MDABR3 cells–derived EVs exhibited increased expression of tight junction proteins compared to those treated with control EVs (Lu et al. 2020). These findings indicate that EVs from highly metastatic BC cells may compromise the BBB and enable cancer cells to cross it, primarily by transferring lncRNA GS1‐600G8.5 (Lu et al. 2020).

Kinjyo et al. (2019) reported that in vitro the EVs released by the B‐cell precursor acute lymphoblastic leukaemia (BCP‐ALL) cell line disrupted the integrity of the BBB. He co‐cultured the BCP‐ALL cell‐secreted EVs with mouse astrocytes (MA cell line) and mouse brain endothelial cells (bEnd.3 cell line) and found that the astrocytes and endothelial cells take them up. He further observed that after incubation with BCP‐ALL cell‐secreted EVs, the MA cells showed a higher level of vascular endothelial growth factor A (VEGF A) at the protein and mRNA levels. Astrocytes‐derived VEGF A has been reported to drive the BBB disruption in the CNS inflammatory diseases (Argaw et al. 2012; Chapouly et al. 2015) and CNS leukaemia (Münch et al. 2017). Kinjyo et al. (2019) further showed that the MA cultured with BCP‐ALL cell‐secreted EVs produced VEGF A and promoted BCP‐ALL cell transmigration across endothelial cell monolayers. He further demonstrated engraftment of BCP‐ALL NALM6 leukaemic cells in NSG female mice resulted in compromised BBB integrity, as evidenced by increased Evans Blue dye incorporation in the brains of NALM6‐engrafted mice compared to controls following intravenous dye injection (Kinjyo et al. 2019).

Elevated mRNA levels of tubulin tyrosine ligase‐like 4 (TTLL4) are significantly associated with shorter recurrence‐free survival and an increased likelihood of BM in BC patients (Arnold et al. 2020). Arnold et al. (2020) reported that TTLL4 overexpression in BC cells (MDA‐MB231 and MDA‐MB468 cells) promotes the biogenesis and secretion of EVs. These MDA‐MB231 and MDA‐MB468 cells–derived EVs then increase the permeability of BBB endothelial (hCMEC/D3) cells and enhance their adhesion to MDA‐MB231 and MDA‐M468 (Arnold et al. 2020).

## 6 Extracellular Vesicles Play an Important Role in Immune Modulation in Brain Metastasis

The miR‐19a in Astrocyte‐released EVs makes BC brain metastatic cells (MDA‐MB‐231, HCC1954, BT474 and MDA‐MB‐435) recruit metastasis‐supportive immune myeloid cells into the tumour microenvironment (Zhang et al. 2015). Adaptive PTEN loss in brain metastatic tumour cells (MDA‐MB‐231, HCC1954, BT474 and MDA‐MB‐435) due to the uptake of miR‐19a‐carrying EVs from primary astrocytes results in a heightened cytokine CCL2 release from them (Zhang et al. 2015). This increase in CCL2 secretion is pivotal in attracting Iba1‐positive myeloid cells to the tumour site (Zhang et al. 2015; Qian et al. 2011). Once recruited, these myeloid cells actively contribute to the progression of BM (Figure 2) (Zhang et al. 2015; Qian and Pollard 2010; Kim et al. 2009) by fostering an environment that supports the tumour cells' rapid proliferation and by inhibiting the natural process of cell death in vivo (Zhang et al. 2015). This reciprocal relationship between the myeloid cells and the tumour cells sustains and accelerates the growth and survival of the brain metastatic tumour cells, thus exacerbating the disease (Zhang et al. 2015).

The loss of X inactive–specific transcript (XIST) has a more pronounced effect on metastasis in the brain than in other organs, as observed in clinical settings and confirmed in xenograft models (He et al. 2006). This suggests that the brain's unique microenvironment, particularly the presence of microglia, influences the colonization abilities of cells with low XIST expression (He et al. 2006). Since microglia typically suppress BM in lung cancer, it is plausible that cells with low XIST expression might re‐programme microglia towards a pro‐metastatic phenotype via cell–cell communication (He et al. 2006). Xing et al. (2018) demonstrated that conditioned medium from MCF7 cells (human breast carcinoma cell line) with silenced XIST expression promoted a shift from M1 to M2 microglia (Figure 2), indicated by changes in CD86 and ARG1 gene expression. This effect was diminished by removing EVs, underscoring their significant role in this conversion. It was further demonstrated that miR‐503 was one of the most prevalent miRNAs in EVs from XIST‐suppressed MCF7 cells (Xing et al. 2018). EVs from MCF7 cells engineered to suppress miR‐503 showed reduced ability to alter microglial phenotype, confirming miR‐503's role in promoting the M1 to M2 transition (Xing et al. 2018). This transition appears mediated by crucial signalling pathways, including STAT3 and the nuclear factor‐kappa B (NFκB) (Wang et al. 2014). In vitro, it was shown that miR‐503 substantially enhances the phosphorylation of STAT3 and reduces the phosphorylation of the p65 subunit of NF‐κB, indicating a systematic re‐programming of the M1 and M2 pathways by miR‐503 (Xing et al. 2018). The conversion of M1 to M2 facilitated by miR‐503 was significantly inhibited by the STAT3 inhibitor, STATTIC (Xing et al. 2018). Additionally, tumour‐associated M2 microglia, similar to M2 macrophages, may promote tumour progression by secreting immunosuppressive factors like PD‐L1 (Prima et al. 2017), which they observed increased in microglia and correlated with suppressed T‐cell proliferation in their models. This points to a complex interaction where tumour‐derived exosomal miR‐503 enhances microglial PD‐L1 expression, dampening local immune responses and promoting tumour growth.

Melanoma BM triggers astrogliosis (Figure 2) and neuroinflammation, which activate pro‐inflammatory responses in the brain metastatic environment (Schwartz et al. 2016). Gener Lahav et al. (2019) explored in vitro that the EVs derived from melanoma played a role in neuroinflammation by investigating their influence on astrocyte activation. Activated astrocytes mediate neuroinflammation (Schwartz et al. 2016; Gilbert and O'Leary 1975) and recruit immune cells in brain conditions, including metastases and autoimmunity (Kim et al. 2014; Placone et al. 2016). In vitro, when primary mouse astrocytes (PMAs) were exposed to EVs derived from mCherry‐expressing Ret melanoma cells (RMCs), qPCR analysis revealed a marked increase in the expression of various pro‐inflammatory cytokines and chemokines, confirming a pro‐inflammatory activation of astrocytes (Gener Lahav et al. 2019). Further protein‐level analysis via a cytokine array supported these findings, indicating RMC‐derived EVs promote pro‐inflammatory signalling in PMA (Gener Lahav et al. 2019). Additionally, it was assessed that this pro‐inflammatory activation could enhance the astrocytes' ability to attract immune cells (Gener Lahav et al. 2019). Migration assays with bone marrow–derived neutrophils and monocytes demonstrated that PMA treated with RMC‐derived EVs had a significantly higher capacity to recruit monocytes (Gener Lahav et al. 2019). These results suggest that melanoma cells can manipulate the metastatic microenvironment by secreting EVs, promoting neuroinflammation and potentially facilitating further metastatic progression (Gener Lahav et al. 2019).

The EVs released from BC can stimulate the NK‐κB activation in macrophages in distant organs, that is, lungs and brain, producing proinflammatory cytokines, that is, interleukin‐6 (IL‐6) and tumour necrosis factor‐α (TNF‐α) (Chow et al. 2014). Mouse experiments in vivo demonstrated that lung and brain macrophages effectively absorb BC‐EVs injected intravenously (Chow et al. 2014). This absorption was associated with an increase in the expression of inflammatory cytokines (Chow et al. 2014). It was further shown that palmitoylated proteins on the surface of BC‐secreted EVs stimulate the Toll‐like receptor 2 (TLR2) on the surface of macrophages and the NF‐κB pathway (Chow et al. 2014). These results highlight the mechanism by which BC induces pro‐inflammatory activity in distant organs' macrophages during its progression to them via EVs (Chow et al. 2014).

The Annexin A2 on the surface of BC EVs (EV‐AnxA2) promotes BC metastasis to the brain in vivo (Maji et al. 2017). Annexin A2 (AnxA2) is a calcium‐dependent phospholipid‐binding protein found in association with the plasma membrane and the endosomal system (Rescher et al. 2008; Valapala and Vishwanatha 2011). It is known to be overexpressed in BC (Maji et al. 2017) and plays roles in several cancer‐related functions (Zhang et al. 2009; Mahdi et al. 2020; Myrvang et al. 2013; Sharma et al. 2016). These include activating plasminogen, re‐arranging the actin cytoskeleton (Rescher et al. 2008) and enhancing cellular migration, adhesion and proliferation (Lokman et al. 2011). EV‐AnxA2 activates macrophages via activating STAT 3 and p38‐NF‐κB pathways (Maji et al. 2017). The activated macrophages release IL‐6 and TNF‐alpha, promoting the formation of a PMN (Maji et al. 2017).

Xu et al. (2023) studied the role of long intergenic non‐coding RNA 00482 (LINC00482) in microglial M2 polarization and promotion of lung cancer BM. LINC00482 was identified as being enriched in serum‐derived EVs of NSCLC BM (Xu et al. 2023). LINC00482 in EVs‐derived lung adenocarcinoma cell lines A549 was found to induce M2 polarization of the microglial cell line (HMC3) in vitro (Xu et al. 2023). It competitively binds to miR‐142‐3p, upregulates the miR‐142‐3p target gene TGF‐β1 and promotes M2 polarization in HMC3 cells (Xu et al. 2023). LINC00482‐induced M2 HMC3 microglia enhanced the malignant properties of A549 cells (Xu et al. 2023). In vivo data in BALB/c nude mouse model showed that EVs transmitted LINC00482, regulating the miR‐142‐3p/TGF‐β1 axis, inducing M2 polarization of microglia and affecting the PMN, thereby promoting BM of NSCLC (Xu et al. 2023). Gan et al. (2020) also reported the effect of EVs derived from lung cancer A549 cell lines (LC‐EVs) on microglial M2 polarization. In vitro, he demonstrated that brain endothelial cells (bEnd.3 cells), upon internalizing the A549 cell‐EVs, release dickkopf‐1 (Dkk‐1). The Dkk‐1, targeting the AMPK pathway, induces pro‐metastatic microglial M2 polarization in the PMN of the brain, promoting cancer metastasis (Gan et al. 2020). EVs derived from BMets_1 cells (surgically collected from a patient with NSCLC BM) carrying miR‐21 induce M2 polarization when taken up by macrophages (monocytic cell line THP‐1 cells) (Tiong et al. 2023).

## 7 Extracellular Vesicles as Predictive Markers for Brain Metastasis

Cancer‐derived EVs carry DNA, RNA and proteins characterizing the tumours (Izhar et al. 2024; Izhar et al. 2024). The transcriptomic and proteomic profiling of cancer‐specific EVs can predict and diagnose the BM of the cancer. Li et al. (2023) used liquid chromatography–MS/MS and investigated the plasma EVs' proteomic profiles of 42 patients with metastatic lung cancer. He found that only Mucin 5B (MUC5B) and selectin L (SELL) on the EV surface showed some accuracy in diagnosing BM of lung cancer with a sensitivity of 69.2% and specificity of 73.2%. Moreover, he found that SELL, Desmoglein‐1 (DSG1), and Podocalyxin like (PODXL) proteins could be used as biomarkers for BM in NSCLC patients with a higher diagnostic value (AUC = 0.844). In addition, it was found that only CD9 had the diagnostic potential to detect BM SCLC cells with AUC = 0.889 (Li et al. 2023).

Similarly, Carretero‐González et al. (2022) prospectively analysed plasma from 123 patients with metastatic cancer, including 42 with BM from various cancers, and 31 healthy controls. After isolating EVs and employing mass spectrometry–‐based proteomics for characterization, they found that patients with b BM had lower EV concentrations but higher global protein concentrations. This increased protein concentration was correlated with poorer overall survival. They also examined levels of STAT3 and PDL1 based on the primary tumour subtype, revealing higher EV‐associated STAT3 levels in patients with BC BM. In comparison, those with melanoma BM exhibited lower STAT3 levels but higher PDL1 levels in EVs.

Chen et al. (2021) identified a significant correlation between elevated levels of EV‐associated integrin β3 and poorer overall survival, along with increased intra‐cranial failure after whole‐brain radiotherapy, in a cohort of 75 patients with lung cancer. The integrin β3 may serve as a novel prognostic biomarker for BM. Hoshino et al. (2015) also in his study demonstrated the role of EV integrin β3 in predicting BC BM. Moreover, in a smaller study involving six lung cancer patients (three with BM and three without), EV RNA sequencing was conducted (Wei et al. 2021). Twenty‐two miRNAs with differential expression were identified, notably a significant upregulation of miR‐550a‐3‐5p in the plasma EVs of patients with BM (Wei et al. 2021). This upregulation was further validated using real‐time quantitative PCR (RT‐qPCR) (Wei et al. 2021).

## 8 Opportunities

When targeting EVs to potentially mitigate their role in BM, a primary approach involves inhibiting their biogenesis. Different molecules such as Manumycin A (Datta et al. 2017; Oh et al. 2017), calpeptin (Yano et al. 1993; Atanassoff et al. 2014), Y27632 (Latham et al. 2013; Tramontano et al. 2004), Imipramine (Deng et al. 2017; Kosgodage et al. 2017) and GW4869 (Catalano and O'Driscoll 2020; Cao et al. 2017; Shamseddine et al. 2015; Menck et al. 2017) inhibit the biogenesis of EVs non‐selectively. However, while EVs contribute to pathological processes, they also play essential roles in physiological functions such as stem cell maintenance, tissue repair, immune surveillance and blood coagulation (El Andaloussi et al. 2013; Rashed et al. 2017; Izhar et al. 2025). Thus, a strategy to selectively target EV subpopulations implicated in BM while preserving those involved in essential physiological processes would represent a significant breakthrough in the field of oncology.

Another approach to targeting EVs and disrupting BM is to inhibit their uptake by recipient cells. However, a significant challenge with this strategy is the incomplete understanding of the precise mechanisms governing EV uptake by cells (El Andaloussi et al. 2013). Furthermore, studies indicate that uptake mechanisms vary depending on both the EVs and the recipient cells involved (McKelvey et al. 2015). Despite these challenges, some inhibitors of EV uptake have been identified. For instance, diannexin disrupts EV uptake by blocking surface phosphatidylserine (PS) (Al‐Nedawi et al. 2009), a key molecule involved in cell adhesion (Lima et al. 2009). However, PS is not exclusive to EVs and is also present on apoptotic cells and activated or angiogenic endothelium, limiting the specificity and broader applicability of this inhibitor. Another example is heparin, which competes with exosomes for binding to cell‐surface heparan sulphate proteoglycans (HSPGs), thereby reducing their uptake (Atai et al. 2013; Christianson et al. 2013). These inhibitors highlight potential pathways for intervention, though further research is needed to overcome limitations such as EV specificity and mechanistic variability.

Moreover, EVs have been engineered as innovative drug delivery vehicles, offering distinct advantages over conventional delivery systems (Imafuku and Sjoqvist 2020). Their stability in blood, small size and ability to evade lysosomal degradation enable them to efficiently deliver therapeutic cargo directly to recipient cells (Ha et al. 2016). Being derived from the patient's own biological material, EVs are less likely to provoke a strong immune response, enhancing their safety profile (Zhang et al. 2022). EV membranes can also be modified with specific proteins or peptides to facilitate targeted interactions with cellular receptors or ECM components (Hwang et al. 2019; Antes et al. 2018), improving their precision in delivering therapeutics to metastatic brain tissue.

EVs also hold significant potential in the diagnosis, prognosis and prediction of BM. By analysing the molecular cargo of EVs, such as proteins, miRNAs, cfDNA and RNAs, they can help identify tumour type and aggressiveness, offering insights into the likelihood of BM development as discussed above. EVs can be isolated from blood, cerebrospinal fluid and urine, providing non‐invasive biomarkers for early detection and monitoring. Their content may reflect disease progression and response to treatment, making them valuable for prognosis. However, further research and standardization are required to validate their clinical utility and integrate EV‐based diagnostics into routine practice.

## 9 Potential Limitations in Current Research

Despite significant advancements in understanding the role of EVs in BM, several limitations in the current body of research must be acknowledged. A significant limitation of past research lies in its predominant focus on EVs, often overlooking the role of non‐vesicular nanoparticles (NVNPs), such as exomeres, supermeres, vaults, lipoproteins, viral particles and albumin. Recent advancements in techniques and methodologies have revealed that NVNPs also carry proteins and nucleic acids, expanding their functional significance in diseases (Zhang et al. 2018; Zhang et al. 2021; Jeppesen et al. 2019; Zhang et al. 2019; Van Niel et al. 2022). For instance, Zhang et al. (2021) demonstrated that supermeres are highly enriched with cargos associated with various cancers (e.g., glycolytic enzymes, TGFBI, miR‐1246, MET, GPC1 and AGO2), Alzheimer's disease (APP) and cardiovascular conditions (ACE2, ACE and PCSK9). Remarkably, the study also highlighted that most extracellular RNA is linked to supermeres rather than small EVs or exomeres (Zhang et al. 2021). Notably, supermeres exhibit the unique ability to traverse the BBB and are preferentially taken up by the brain (Jeppesen et al. 2023), suggesting their potential as carriers of therapeutic agents for brain disorders and as vehicles for understanding CNS‐related pathologies. Similarly, Zhang's discovery of exomeres revealed their unique N‐glycosylation patterns, protein, lipid, DNA and RNA profiles, as well as distinct biophysical properties compared to exosomes (Zhang et al. 2018).

One of the primary challenges has been the limited availability of robust techniques and methodologies to effectively isolate EVs from NVNPs (Zhang et al. 2023). Conventional EV isolation methods, such as differential ultracentrifugation, size‐exclusion chromatography and precipitation‐based techniques, often result in co‐isolation of NVNPs due to overlapping physical properties like size and density (Zhang et al. 2023). This lack of specificity has hindered the ability to study the distinct functions and mechanisms of EVs and NVNPs separately. Historically, this methodological limitation might have led to the misattribution of certain biological functions exclusively to EVs, while the contributions of NVNPs remained unrecognized. Consequently, our understanding of extracellular particle‐mediated mechanisms in various diseases, including cancer and BM, has been incomplete. The presence of NVNPs not only challenges established EV‐driven pathways but also underscores the urgent need for improved isolation and characterization techniques to delineate the unique roles of NVNPs in disease processes. Therefore, integrating NVNPs alongside EVs in future research is crucial to uncovering their mechanisms and diagnostic potential in different diseases.

Another major limitation lies in the heavy reliance on cell lines and animal models, which, while invaluable for elucidating EV mechanisms, often fail to replicate the complexity of human biology. Studies using cell lines provide controlled environments to investigate processes such as BBB disruption and PMN formation; however, these models lack the genetic and phenotypic heterogeneity observed in human tumours. Furthermore, prolonged culture can lead to genetic drift, potentially skewing results. Similarly, animal models, particularly murine systems, are essential for studying EV dynamics in vivo but are limited by interspecies differences in immune responses, BBB structure and brain microenvironment composition. These discrepancies reduce the translatability of findings to human patients, especially since immune‐deficient models oversimplify the intricate immune interactions seen in clinical settings. To address these challenges, future research should prioritize clinically relevant approaches, such as using patient‐derived organoids or xenografts that better mimic the human tumour microenvironment. Additionally, longitudinal studies are needed to validate the diagnostic and prognostic potential of EVs in BM.

Investigations should prioritize exploring the interactions of EVs and NVNPs with human‐specific factors, such as the immune system, PMN and the BBB, to achieve a more precise and comprehensive understanding of their role in brain metastasis. Addressing these critical gaps will pave the way for translating EV‐ and NVNP‐based discoveries into innovative diagnostic and therapeutic strategies for BM. Despite the pressing need, research on BM remains limited, resulting in significant gaps in understanding the roles of both EVs and NVNPs in this context. Further studies are essential to unravel their mechanisms of action, define their contributions to disease progression and develop more effective therapeutic interventions and highly sensitive diagnostic biomarkers. By bridging these gaps, the immense potential of EVs and NVNPs as tools for understanding and treating BM can be fully harnessed.

## 10 Conclusion

EVs are useful for diagnostic and prognostic purposes and play significant roles in cancer pathogenesis and metastasis. EV miRNAs and proteins contribute to vascular disruption, angiogenesis, cancer cell progression, PMN formation, immune modulation within the PMN and metastasis. This review discusses how EVs facilitate BM by forming PMNs, modulating the immune system, and compromising the BBB integrity. These biomarkers can also provide important information regarding the metastatic potential of cancer before metastasizing to the brain. Understanding these mechanisms highlights the potential of targeting EVs in therapeutic strategies against BM.

## Author Contributions

**Muhammad Izhar**: conceptualization, data curation, formal analysis, methodology, software, validation, writing–original draft, writing–review and editing. **Maciej S. Lesniak**: conceptualization, data curation, formal analysis, funding acquisition, methodology, project administration, resources, software, supervision, validation, visualization, writing–original draft, writing–review and editing.

## Conflicts of Interest

The authors declare no conflicts of interest.
